# Prone versus supine radiotherapy for right-sided breast cancer – a retrospective study of radiotherapy plans

**DOI:** 10.1186/s13014-026-02884-z

**Published:** 2026-07-15

**Authors:** Gunnar Lohm, Konrad Neumann, Nina Thieß, Daniel Medenwald, Evelyn Weinstrauch

**Affiliations:** 1 Department of Radiation Oncology, Johanniter-Hospital Stendal, 39576 Stendal, Germany; 2 Department of Radiation Oncology, Charité Universitätsmedizin, 13353 Berlin, Germany; 3 Institute of Biometry and Clinical Epidemiology, Charité Universitätsmedizin, 10117 Berlin, Germany; 4 University Clinic for Hematology, Oncology, Cell Therapy and Radiation Therapy, Department for Radiation Oncology, Otto von Guericke University, 39120 Magdeburg, Germany

**Keywords:** Breast cancer radiotherapy, Prone position, Deep inspiratory breath hold, Planning study

## Abstract

**Background:**

In patients with lumpectomy for left-sided early breast cancer, whole breast irradiation (WBI) in prone position (PP) is an acceptable alternative to WBI in supine position (SP). We compared the less well explored right-sided PP WBI with SP WBI to identify the optimal organ at risk protection.

**Methods:**

We retrospectively analyzed WBI-plans for 30 patients with right-sided early breast cancer in PP and SP. SP-plans were created with intensity-modulated radiotherapy (IMRT) and deep inspiratory breath hold (DIBH); PP-plans with 3D conformal radiotherapy and free breathing (FB). For better comparison of the different positioning, we additionally calculated IMRT-plans in PP and FB. We compared the extent of the clinical and planning target volumes (CTV and PTV) and the radiotherapy doses applied to CTV, PTV, right lung, heart, coronary arteries, left breast, and the right-sided axillary levels. Furthermore, we analyzed the possible impact of the breast size on the doses applied to the heart and right lung.

**Results:**

Compared to SP, PP WBI was associated with lower doses applied to the right lung (Dmean right lung SP 5.6 Gy vs. 3.5 Gy PP, *p* < 0.001) but higher doses to the heart (Dmean heart SP 0.4 Gy vs. 0.7 Gy PP, *p* < 0.001) and coronary arteries, and a diminished dose coverage of the target volumes (V95_PTV_ PP 90.4% vs. 92.9% SP, *p* < 0.001). We found no statistically significant association between the CTV volume and the mean doses applied to the heart, right ventricle, right coronary artery, and right lung.

**Conclusions:**

In patients scheduled for WBI in PP, the advantages for the right lung should be weighed against the cardiovascular disadvantages. However, the doses applied to both organs are not only related to the patient´s positioning but also to the used technique and could be reduced by employing a DIBH-technique in PP. A statistically significant relationship was not found between breast size and positioning suitability, but the study may be limited in power to detect small differences.

## Background

Following breast-conserving cancer surgery, adjuvant radiotherapy halves the recurrence rates and reduces the breast cancer-specific death rate [1]. However, whole breast irradiation (WBI) increases the risk of cardiac morbidity and mortality, and therefore, all measures reducing cardiac exposure should be attempted [2]. Furthermore, WBI is associated with an increased risk for radiation-induced pneumonitis [3] and secondary cancers [4]. Facing these sequelae associated with WBI, several studies documented reduced doses applied to the heart and lung by implementing the technique of deep inspiratory breath hold (DIBH) for adjuvant irradiation in breast cancer patients [5–7]. Hence, DIBH should be considered the standard of care for WBI in the supine position (SP).

In 1994, WBI in the prone position (PP) as an alternative technique for irradiating the breast following breast-conserving surgery has been described. This approach was considered as an effective alternative to WBI in conventional SP when irradiation in SP is likely to result in unacceptable dose inhomogeneities within target volumes or enhanced doses applied to adjacent normal tissues [8]. In a subsequent study of 11 patients with large pendulous breasts, an incomplete coverage of the minimal target volume consisting of the biopsy cavities with a margin of 2 cm was found in 73% of the patients, especially when the biopsy cavity was located close to the chest wall [9]. During the following decades, WBI in PP was increasingly adopted due to the implementation of commercially available devices providing a stable and reproducible positioning of the patients in PP [10, 11] and increasing numbers of studies evaluating the advantages of this radiation technique [12]. In the same period, hypo-fractionated radiotherapy after breast conserving surgery [13] increasingly became the standard of care, especially, after the benefits of this approach were confirmed by meta-analyses of subsequently performed trials [14, 15]. At present, the German S3 guideline proposes the hypo-fractionated radiotherapy for WBI in 15 or 16 fractions with a total dose around 40 Gy (Gy) as the first choice option, in patients without lymph node involvement [16].

The majority of studies exploring WBI in PP were performed for left-sided breast cancer. A PubMed search for clinical trials with the keywords “breast cancer radiotherapy prone” and “left” revealed 16 results compared to 6 results when added the word “right” instead of “left”. In view of limited available data about adjuvant radiation of patients with right-sided, early breast cancer in PP, we evaluated the effects of hypo-fractionated WBI in PP in this particular patient subgroup in order to identify an optimal protection of adjacent organs at risk as well as optimal target coverages. Furthermore, we tested the hypotheses from previous publications that PP WBI is only suitable for women with larger breasts. Here, we present our planning experiences and the results of our analysis comparing WBI in PP and free breathing (FB) with WBI in SP and DIBH.

## Methods

### Study design

After approval of the Ethics Committee of the Ärztekammer Sachsen-Anhalt (file reference number: 35/22), we retrospectively analyzed all radiotherapy plans from a subgroup of 30 patients with dual planning in PP and SP among a total of 319 patients with a completed WBI treatment at our department between June 2020 and September 2025 and who had undergone a lumpectomy for right-sided early breast cancer without lymph node metastases or with breast cancer in situ (pTis). We offered the different treatment positions, including the dual planning, to all patients except for those with simultaneous contralateral breast cancer. This was not done according to a specific protocol or a pre-structured information process. Reasons for choosing SP or PP only were: SP (n = 172) due to simultaneous left-sided breast cancer and patient´s choice (better protection of the heart and/or simultaneously integrated boost requiring IMRT), PP (n = 117) due to patient´s choice (better protection of the lung). Dual planning was performed in 30 patients to identify the optimal approach for heart and lung protection. All plans were calculated for a total dose of 42.56 Gy in 16 single fractions of 2.66 Gy. For all patients, we established radiotherapy plans for both positions: SP with IMRT in DIBH and in PP with 3D conformal plans and FB. We then selected those plans for therapy of individual patients, which provided better protection of the organs at risk (OAR). For better comparison with previous studies, we additionally calculated IMRT plans with FB in PP.

The computed tomography (CT) scans were performed with a Somatom Definition AS CT scanner (Siemens Healthineers, Forchheim, Germany) in 3–5 mm slices. DIBH was realized with the Respiratory Gating for Scanners (RGSC) System (Varian Medical Systems, Palo Alto, CA, USA) with the marker block placed on the epigastrium. The PP was realized with the Sagittilt device (Orfit Industries, Wijnegem, Belgium), routinely tilted ten degrees to the right side, which to our experiences provided a reasonable compromise between the patient´s comfort and the desired lateralization of the breast for better radiotherapy planning. To further enhance the patient´s comfort, we applied a backside mask. We established all radiotherapy plans with Eclipse version 13.6 for a True Beam linear accelerator with 120 multileave collimator version 2.7 of Varian Medical Systems (Palo Alto, CA, USA). 

### Target volumes

**Fig. 1:**
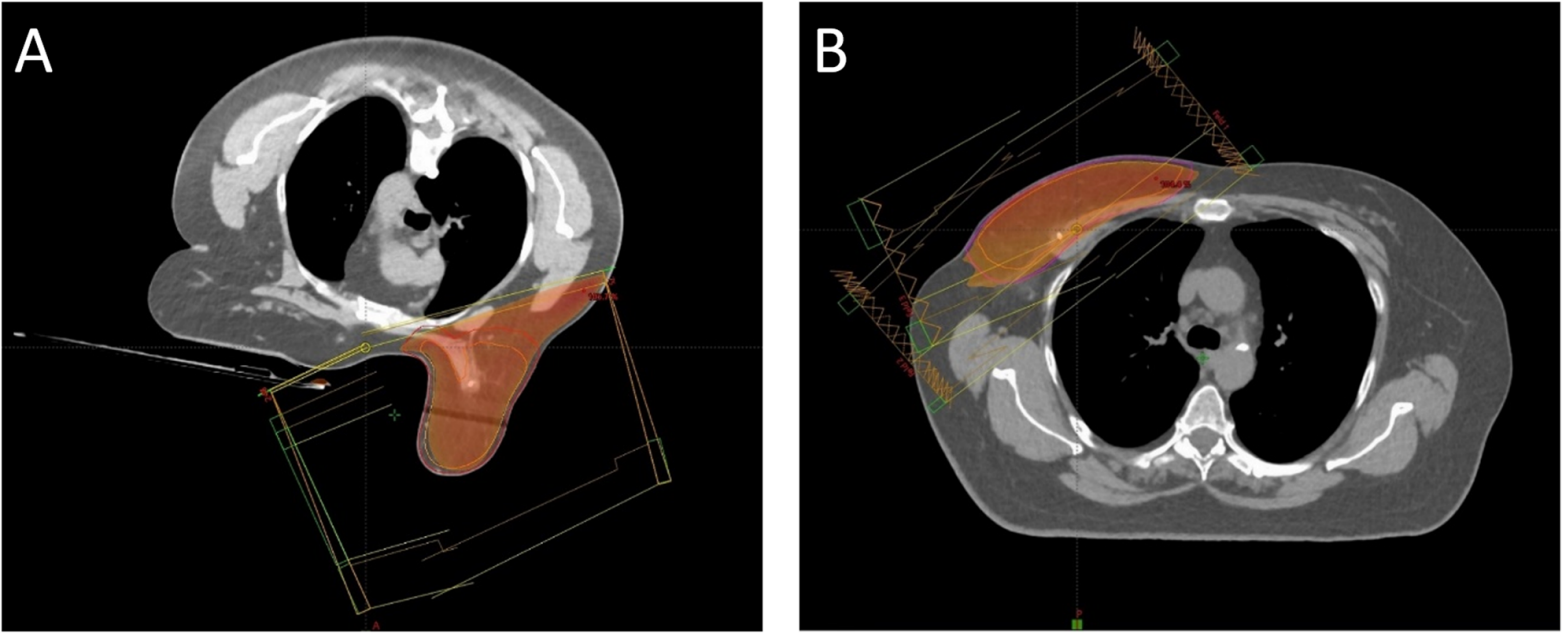
CT-scan of a patient from the investigated cohort. (**A**) Prone position; (**B**) Supine position. Note: The orange line indicates clinical target volumes (CTV), and the red line indicates planning target volumes (PTV). The orange area represents the applied dose of the radiation plan. White lines indicate irradiation-fields

**Fig. 2:**
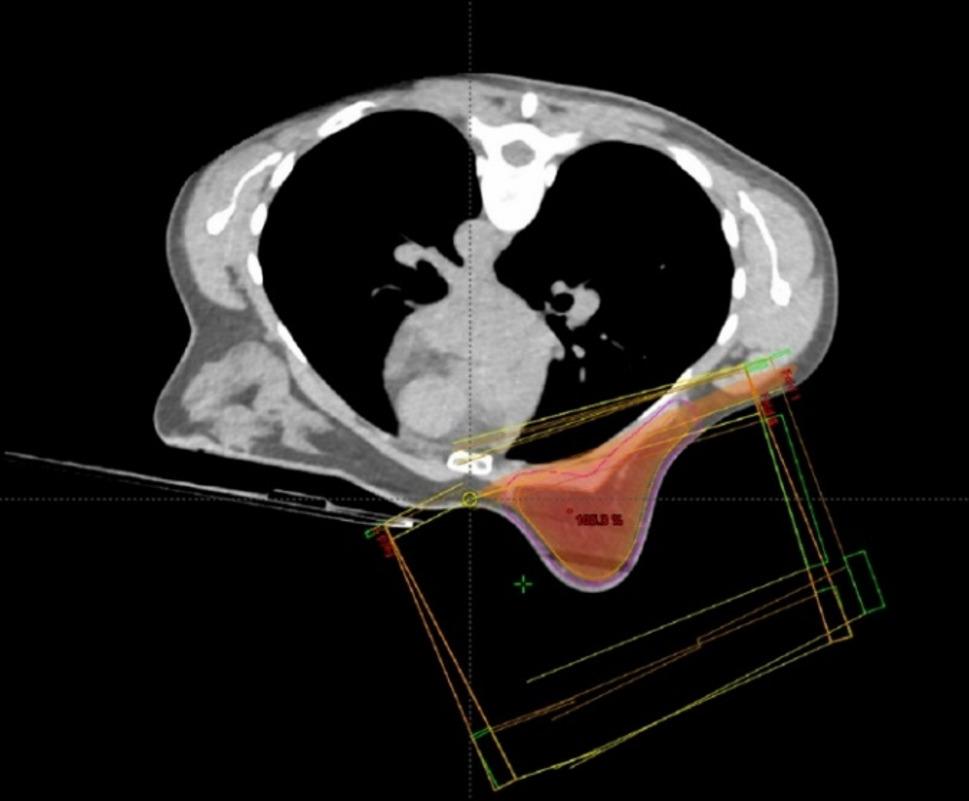
CT-scan from the investigated cohort with a smaller breast size in the prone position. Note: The orange line indicates clinical target volumes (CTV), and the red line indicates planning target volumes (PTV). The orange area represents the applied dose of the radiation plan. White lines indicate irradiation-fields

Clinical target volumes (CTV) for the supine position were delineated according to the consensus guideline of the European Society for Radiotherapy and Oncology (ESTRO) [17]. As described in the ESTRO guideline, we reduced the CTV from the skin surface by 5 mm. For delineation of the CTV in PP, no consensus guideline exists. Hence, we delineated the CTV similarly. We added a margin of 6 mm to the CTV for defining the planning target volume (PTV) in PP, according to the results of a previously published study where the same prone positioning Sagittilt device was used as in the present study. In that study, a ≥5 mm CTV-PTV margin was proposed due to a lateral/longitudinal/vertical systematic and random residual-intrafractional error of 1.5/0.9/1.7 mm and1.7/1.9/1.6 mm, requiring a CTV–PTV margin of 5.0/3.6/5.4 mm, respectively [11]. For SP, we used the same CTV-PTV margin, which was a single mm wider than the previously proposed margin of 5 mm [18]. PTVs were reduced from the skin surface by 2 mm. The delineation of CTV (orange line) and PTV (red line) in prone and supine position are displayed in Fig. 1 and (only in prone) in Fig. 2. 

Furthermore, the example in Figure 2 highlights the impact of a small breast size on the shape of the breast in PP, with almost no tenting of the major pectoral muscle.

### Organs at risk

We delineated the left breast, lungs, heart, ventricles, left anterior descending artery (LAD), and right coronary artery (RCA). The structures of the heart were segmented according to the cardiac contouring atlas of Duane et al. [19]. For quality assurance, the delineation of all heart structures was checked by two skilled radiotherapists who strictly followed the recommendations of the heart atlas presented in the supplement of the publication of Duane et al. [19]. Doses applied to the heart and its substructures were limited according to proposed constraints [20]: D_mean_ heart < 2.5 Gy, D_max_ LAD < 10 Gy, V30_LAD_ < 2%, V40_LAD_ < 1%, D_mean_ left ventricle (LV) < 3 Gy, V5_LV_ < 17% and V23_LV_ < 5%. To our best knowledge, commonly accepted dose constraints for the right ventricle and the RCA do not exist. Hence, we chose the corresponding constraints for the left-sided substructures of the heart as surrogate constraints. For the right lung, D_mean_ had to be < 10 Gy (target goal < 7 Gy). 

### Axillary levels

We delineated the axillary levels I to III in SP and PP according to the current guideline of the Radiation Therapy Oncology Group (RTOG) [21]. For comparison with the literature, we additionally delineated the axillary levels I and II in PP according to a recently published proposal [22]. 

### Technical realization of radiation plans

We performed all planning using 6 and 15 MV photon energies. For 3-dimensional (3D) PP plans, two main opposing fields were created with angles between 70° and 90° for the lateral field and 235° and 265° for the opposing medial field. These fields were shaped with wedge filters and complemented with supplementary fields angled +/- 10° in reference to the main fields with or without additional wedge filters, according to the requirements of target coverage and dose homogeneity. These fields were enlarged with margins between 15 and 30 mm beyond the skin surface to cover potential breast swelling during radiotherapy and uncertainties of the breast positioning. For SP, we used an IMRT. Field angles of 40° to 65° were chosen for the medial field and 220° to 240° for the lateral field. In analogy to PP, fields were enlarged by 15 to 30 mm using the Skin Flash function of the planning system for field enlargement. We created all radiotherapy plans to achieve the prescribed mean PTV dose of 42.56 Gy. For better comparison with previously published analyses, we additionally created IMRT in PP (IMRT PP), which we did not apply to our patients as outlined in the discussion section. The field angles for these IMRT-plans ranged from 70° to 90° for the lateral field and 230° to 265° for the medial field. 

### Statistical analyses

After testing for normal distribution by using the Kolmogorov-Smirnov-test, we performed two-sided t-tests for paired samples to compare the investigated parameters in PP versus SP. A potential association of the breast size represented by the CTV volumes with doses applied to the heart, right ventricle, right coronary artery, and right lung was investigated with a linear regression analysis.

All calculations were performed with IBM SPSS Statistics version 29 (IBM Corporation, Armonk, NY, USA). 

### Estimation of the cardiovascular risk and the risk of secondary lung cancer due to WBI

We calculated the risk of inducing coronary events or secondary lung cancer according to previously published estimations on the dose-dependent impact on the heart [23, 24] and the lungs [25, 26]. 

## Results

Overall, data from 30 patients with dual plannings in PP and SP were available, and there was a wide range concerning age and body mass index (Table 1).

**Table 1:** Baseline patient and tumor data (*n* = 30)

Variable/Characteristic	Value
**Continuous variable**	
Age at start of radiotherapy (years), median (range)	62.7 (40.8–78.4)
Body weight (kg), median (range)	72.0 (43–100)
Body mass index (BMI)^1^, median (range)	25.7 (19–35.4)
**Tumor characteristic**	
Pathological T stage (pT), n	
pTis	3
pT1a	2
pT1b	4
pT1c	10
pT2	10
pT3	1
UICC stage, n	
0	3
IA	16
IB	0
IIA	9
IIB	2

Note: Continuous variables are presented as median (range), categorical variables as n. ^1^BMI = body weight/(body height)^2^

Among these 30 patients, six individuals were active smokers with 25–40 packyears and one patient had a history of smoking until 15 years prior to this study (30 packyears).

Comparison of PP vs. SP revealed significantly larger CTVs and PTVs in PP compared to SP (*p* < 0.001 for both parameters; as illustrated in Figs. 1 and 2). Furthermore, we detected a small but significantly reduced target coverage for the plans in 3D-PP compared to SP (mean D90: PP 40.5 Gy vs. SP 41.0 Gy, difference 1.2% and mean V95 PP 90.4% vs. SP 92.9%, difference 2.7%, *p* < 0.001 for both parameters: Table 2). In the extra-analysis of PP IMRT, the mean D90 and V95 were higher compared to the 3D-plans, and compared to SP, the D90 PP IMRT was not significantly reduced (*p* = 0.067). 

**Table 2:** Target volume and coverages

Volume/Coverage	Supine mean (SD)	Prone mean (SD)	*p*-value^1^ (supine vs. prone)
CTV (mL)	609.6 (296.1)	689.1 (361.4)	< 0.001***
PTV (mL)	877.5 (348.5)	925.1 (429.7)	< 0.001***
D_max_PTV (Gy)	45.8 (0.42)	3D: 45.7 (0.41)	3D: 0.629
		IMRT: 45.9 (0.45)	IMRT: 0.230
D90 PTV (Gy)	41.0 (0.37)	3D: 40.5 (0.46)	3D: <0.001***
		IMRT: 40.8 (0.37)	IMRT: 0.067
V95 PTV (%)	92.9 (1.88)	3D: 90.4 (2.05)	3D: <0.001***
		IMRT: 91.9(1.80)	IMRT: 0.011*

Note: ^1^2-sided *t*-test for paired samples. *p < 0.05, ***p < 0.001. Abbreviations: SD, standard deviation; CTV, clinical target volume; PTV, planning target volume; IMRT, intensity-modulated radiotherapy

The analyses of the doses applied to the right lung showed significantly lower values for D_mean_, V5, V20, and V30 in 3D-PP compared to SP(e.g., D_mean_ PP 3.5 Gy vs. SP 5.6 Gy, *p* < 0.001), whereas D_max_ to the right lung was lower in SP. The corresponding analysis of PP IMRT revealed even lower doses applied to the right lung.

The findings of the dose parameters for the heart, including the ventricles and the coronary arteries, were all significantly lower in SP (e.g., D_mean_ heart PP 0.7 Gy vs. SP 0.4 Gy, *p* < 0.001) except for V30 to the heart and D_max_ applied to the LAD, which showed no significant difference when compared between both positions The mean Dmax Heart in PP was 16.4 Gy for 3D PP (13.4 for PP IMRT) with a standard deviation of 10.4 Gy, (10.0 for PP IMRT) (Table 3). This indicates a high inter-patient variability with a large range of 2.6–36.5 Gy (2.6–34.9 for PP IMRT).

**Table 3:** Doses applied to the right lung and heart, including the coronary arteries

Dose	Supine mean (SD)	Prone mean (SD)	*p*-value^1^ (supine vs. prone)
D_mean_ Lung (Gy)	5.6 (1.4)	3D: 3.5 (1.3)	3D: <0.001***
		IMRT: 3.0 (1.2)	IMRT: <0.001***
D_max_ Lung (Gy)	41.2 (1.3)	3D: 41.8 (1.2)	3D: 0.007**
		IMRT: 41.0 (1.9)	IMRT: 0.381
V5 Lung (%)	22.8 (6.4)	3D: 13.0 (6.3)	3D: <0.001***
		IMRT: 12.4 (5.5)	IMRT: <0.001***
V20 Lung (%)	10.4 (4.4)	3D: 6.0 (2.8)	3D: <0.001***
		IMRT: 5.2 (3.1)	IMRT: <0.001***
V30 Lung (%)	7.1 (3.8)	3D: 3.7 (2.0)	3D: <0.001***
		IMRT: 1.9 (1.4)	IMRT: <0.001***
D_mean_ Heart (Gy)	0.4 (0.2)	3D: 0.7 (0.3)	3D: <0.001***
		IMRT: 0.6 (0.3)	IMRT: <0.001***
D_max_ Heart (Gy)	4.1 (2.2)	3D: 16.4 (10.4)	3D: <0.001***
		IMRT: 13.4 (10.0)	IMRT: <0.001***
D5 Heart (Gy)	1.3 (1.0)	3D: 2.2 (1.5)	3D: 0.003**
		IMRT: 2.0 (1.3)	IMRT: 0.011*
V30 Heart (%)	0.0 (0.0)	3D: 0.001 (0.005)	3D: 0.326
		IMRT: 0.03 (0.1)	IMRT: 0.258
D_mean_ Ventricle _R_ (Gy)	0.4 (0.4)	3D: 0.6 (0.3)	3D: 0.010*
		IMRT: 0.6 (0.3)	IMRT: 0.080
D_max_ Ventricle _R_ (Gy)	1.6 (1.4)	3D: 2.8 (2.2)	3D: 0.011*
		IMRT: 2.9 (2.8)	IMRT: 0.022*
D_mean_ Ventricle _L_ (Gy)	0.1 (0.06)	3D: 0.2 (0.07)	3D: <0.001***
		IMRT: 0.1 (0.07)	IMRT: 0.005**
D_max_Ventricle _L_ (Gy)	0.4 (0.2)	3D: 0.5 (0.2)	3D: <0.001***
		IMRT: 0.5 (0.2)	IMRT: 0.009**
D_mean_ RCA (Gy)	1.3 (0.8)	3D: 2.5 (2.0)	3D: 0.002**
		IMRT: 2.5 (2.1)	IMRT: 0.005**
D_max_ RCA (Gy)	2.4 (1.7)	3D: 6.1 (6.6)	3D: 0.003**
		IMRT: 6.6 (7.8)	IMRT: 0.005**
D_mean_ LAD (Gy)	0.12 (0.1)	3D: 0.23 (0.1)	3D: <0.001***
		IMRT: 0.19 (0.1)	IMRT: 0.026*
D_max_ LAD (Gy)	0.34 (0.4)	3D: 0.39 (0.2)	3D: 0.598
		IMRT: 0.34 (0.2)	IMRT: 0.999

Note: ^1^2-sided *t*-test for paired samples. *p < 0.05, **p < 0.01, ***p < 0.001. Abbreviations: SD, standard deviation; RCA, right coronary artery; LAD, left anterior descending coronary artery; IMRT, intensity-modulated radiotherapy

The estimation for cardiovascular events after radiotherapy for breast cancer revealed a linear increase of 7.4% per Gy mean dose applied to the heart [23]. In our analysis, the difference in the mean heart doses (Δ 3D-PP – SP) was 0.3 Gy, leading to an excess relative risk for cardiovascular events of 2.22% in patients treated in PP. The absolute risk for ischemic heart disease was previously estimated to be approximately 1/400 [24, 26], leading to an excess in the order of 1 per 20,000 irradiated patients in PP for cardiovascular events.

The risk of acquiring secondary lung cancer due to irradiation for breast cancer was estimated to increase linearly by 8.5% per Gy applied to the lung for non-smokers [26]. In our analysis, the differences of mean lung doses (Δ SP – 3D-PP) were 2.1 Gy. This leads to an increased relative risk for acquiring lung cancer in SP of at least 17.8% compared to PP. The absolute risk for lung cancer due to irradiation in patients with early breast cancer was previously estimated to be 1/233 [25], leading to an excess of 14 per 20,000 irradiated patients in SP for secondary lung cancers.

The analysis of the doses applied to the left breast as an additional organ at risk showed overall higher values in 3D-PP (and IMRT PP) for V5, D_mean,_ and D_max_ compared to SP, but reached no statistical significance (Table 4).

In our analysis of the axillary levels, we found significantly larger level I and II sizes in PP compared to SP, but no significant differences for level III. The investigation of the accidental dose coverage of the axillary levels revealed a significant difference only for level II with a higher D_mean_ applied in SP IMRT compared to 3D-PP and PP IMRT (Table 4).

**Table 4:** Doses applied to the left breast and axillary levels

Dose	Supine	Prone	*p*-value^1^
	mean (SD)	mean (SD)	*(supine vs. prone)*
D_mean_Breast_L_ (Gy)	0.3 (0.3)	3D: 0.43 (0.5)	3D: 0.199
		IMRT: 0.40 (0.6)	IMRT: 0.420
D_max_Breast_L_ (Gy)	7.9 (1.0)	3D: 12.4 (11.8)	3D: 0.077
		IMRT: 8.8 (8.9)	IMRT: 0.654
V5 Breast_L_ (%)	1.0 (2.2)	3D: 2.1 (3.9)	3D: 0.172
		IMRT: 1.9 (4.3)	IMRT: 0.340
Vol. Axilla Level I (mL)	49.7 (16.5)	56.4 (18.6)	0.007**
D_mean_ Level I Gy)	23.9 (7.3)	3D: 26.3 (9.2)	3D: 0.265
		IMRT: 23.1 (8.1)	IMRT: 0.722
Vol. Axilla Level II (mL)	12.8 (5.4)	18.6 (4.8)	< 0.001***
D_mean_ Level Il (Gy)	11.2 (9.0)	3D: 5.9 (4.7)	3D: 0.003**
		IMRT: 4.7 (3.6)	IMRT: <0.001***
Vol. Axilla Level III (mL)	9.4 (3.8)	9.8 (3.2)	0.546
D_mean_ Level III (Gy)	1.8 (2.8)	3D: 0.9 (0.4)	3D: 0.93
		IMRT: 0.8 (0.3)	IMRT: 0.63

Note: ^1^2-sided *t*-test for paired samples. **p < 0.01, ***p < 0.001. Abbreviations: SD, standard deviation; Vol. = Volume; IMRT, intensity-modulated radiotherapy

The linear regression analysis revealed no statistically significant association between the CTV volumes and the investigated parameters (i.e., mean doses applied to heart, right ventricle, right coronary artery, and right lung), neither for 3D-PP nor PP IMRT. The slope of the regression lines (not shown) increased towards larger CTV volumes for the mean doses applied to the heart, right ventricle, and right lung, regardless of the investigated radiotherapy technique in PP. These findings indicated a trend towards higher doses applied to these parameters in women with larger breasts, whereas the analysis for the right coronary artery showed an opposite trend (Table 5).

**Table 5:** Associations between breast size and organ doses applied by the two optimal available radiation strategies

Parameter	*p*-Value for PP 3D	RC (95% CI)	*p*-value for SP IMRT	RC (95% CI)
D_mean_ heart (Gy)	0.471	0.000 (0.000–0.000)	0.592	0.000 (0.000–0.000)
D_mean_ right ventricle (Gy)	0.272	0.000 (0.000–0.000)	0.287	0.000 (0.000–0.001)
D_mean_ right coronary artery (Gy)	0.541	-0.001 (-0.003–0.002)	0.429	-0.001 (-0.004–0.002)
D_mean_ right lung (Gy)	0.649	0.000 (-0.001–0.002)	0.812	0.000 (-0.001–0.002)

Breast size is represented by the clinical target volume in prone position for the analysis in prone and for the corresponding analysis in supine position, respectively

Abbreviations: PP, prone position; 3D, 3 dimensional; SP, supine position; IMRT, intensity-modulated radiotherapy; RC, regression coefficient; CI, confidence interval

## Discussion

There is an ongoing debate about the advantages and disadvantages of WBI in PP compared to SP. For right-sided breast cancer, irradiation in PP has not been extensively explored, and the present study was performed to broaden the available data about this therapeutic option for patients with resected early, right-sided breast cancer.

Delineating the target volumes in PP is challenging, and commonly accepted criteria for delineation of the whole breast in PP for WBI have not been developed. As shown in Figure 1 from the same patient with a surgical clip placed on the ventral surface of the major pectoral muscle, the anatomy of the structures is very different in the prone position when compared to the supine position. The target volume in PP is stretched ventrally, and the major pectoral muscle shows a tenting and no longer covers the minor pectoral muscle.

As outlined, we chose the ESTRO guideline for delineating the CTV and the medial borders and the borders to the major pectoral muscle, as well as to the skin. Using this approach, the CTVs appear to be similar for both positions. However, our CTVs in prone were larger than in SP (see Table 2). This might be explained by different tissue stretching in PP and is in line with a previously published study where larger CTV volumes in PP compared to SP were reported in 63 patients with 33 left-sided and 30 right-sided breast cancers [27].

Our findings showed a slight but statistically significant decrease in coverage of the PTV in 3D-PP compared to SP with IMRT, documented by the diminished mean values for D90PTV and V95PTV in 3D-PP (1.2% and 2.7%, respectively, p<0.001 for both parameters, as outlined in the result section). These findings are in contrast to the results from a previously published study, where no significant differences in the target coverage between PP and SP were observed using 3D conformal radiotherapy for SP and PP (meanD90PTV SP 43.1 Gy vs. PP 42.9 Gy, difference 0.46%, p=0.42 and meanV95PTV SP 90.7% vs. PP 89.3%, difference 1.3%, p=0.29) [28]. In a planning study with 6 right-sided and 12 left-sided breast cancer patients, IMRT provided a superior dose coverage of the PTV compared to a technique using wedged tangential fields in PP with a dose-coverage index (proportion of the planning target volume for optimization covered by the 95–107% range of the prescription dose) for wedged tangential field radiotherapy in PP 97.2% vs. 97.7% for a tangential field IMRT [29]. In our study, we used IMRT only for SP WBI which well explains the diminished dose coverage in PP compared to SP. This suggests that, IMRT should be considered for PP-WBI. However, with our PP setup using the Sagittilt device, we experienced visible changes in breast position during the radiotherapy sessions, depending on the patient´s body tension. Hence, without breathing control and without a surface-guided system, we decided not to implement IMRT for irradiating in PP. In our extra analysis with IMRT PP, we detected a meanD90PTV of 40.5 Gy for 3D PP plans vs. 45.9 Gy for PP IMRT and a meanV95PTV of 90.4% for 3D PP vs. 91.9% for PP IMRT (p<0.001 for both analyses). These findings were in line with the previously published results [29].

Regardless of the chosen technique, the irradiation in PP with FB provided a better protection of the right lung, but inversely resulted in higher doses applied to the heart and its substructures (see Table 3). However, a better protection of the heart and the coronary arteries in PP should be achieved by adding DIBH to radiation in PP which is the standard of care for WBI in SP. This has already been proven superior for left-sided WBI in PP with a DmeanHeart for PP and shallow breathing of 2.2Gy vs. 1.3 Gy for PP and DIBH and a DmeanLAD for PP and shallow breathing of 8.3 Gy vs. 3.3 Gy for PP and DIBH, p<0.001 for both analyses [30]. However, our RGSC-system for breathing control during radiotherapy is restricted to SP, only.

The analysis of the Dmax applied to the heart revealed a large range in PP. These data were in line with a previously published analysis, where a Dmax Heart of 37.48 Gy for larger breasts and smaller breasts of 35.35 Gy (standard deviation 10.48 and 11.89, respectively) was reported without statistically significant difference (p = 0.57) [31]. Furthermore, in that study, the values of standard deviations for Dmax Heart were in the order of our results. A possible explanation for this finding could be a larger variability of the intra-thoracic anatomy in PP, which might lead to an increased rate of radiation-induced cardiac disease to some patients. However, the reason for this a high inter-patient variability of the Dmax Heart and its relevance remained unclear and should be further explored in clinical investigations.

Our estimation of the cardiovascular risk and the risk of secondary lung cancer revealed a superiority of PP-WBI in preventing patients from secondary lung cancers, which exceeds the cardiovascular hazard caused by this radiotherapy technique compared to SP-WBI.

According to a large meta-analysis, the hazard of dying from a breast cancer relapse exceeds by far the risks of dying from myocardial infarction or secondary lung cancer associated with WBI. [1]. Hence, WBI should always provide an optimal coverage of the target volumes, which should not be compromised by attempts to protect the OAR.

Our analysis of the axillary levels revealed enlarged volumes for level I and II in PP. For level I, this enlargement is well explained by stretching of this area in prone, especially towards the anterior surface of the subscapular muscle. For level II, the enlargement could be explained by the different spatial course of the axillary vessel, which is a landmark for the delineation of this level. The accidental coverage of the axillary levels in the present study were not significantly different for PP compared to SP, except for level II. For that level, the applied dose was diminished in PP (Dmean Level II SP 11.2 Gy vs. 5.9 Gy in PP, p=0.003, see Table 4), which is in line with a previously published study. In that investigation, the Dmean for Level II, calculated in % of the prescribed dose, was SP 6% vs. 3% in PP [32]. Additionally, we delineated the axillary levels I and II in PP according to the recently published proposal of Purswani et al. [22]. Due to the expansion of the level I towards the skin and the different boundaries of level II, this delineation method resulted in further enlarged volumes compared to a delineation according to RTOG guidelines (Mean volume Level I 79.2 mL [22] vs. 56.4 mL [RTOG]) and mean volume Level II 21.2 mL [22] vs. 12.8 mL [RTOG]). Due to these findings, a direct comparison of the doses applied to the axillary levels in PP vs. SP I is largely imprecise when using this novel delineation guideline.

Our finding of a diminished dose coverage of Level II in PP might result in an enhanced risk for a nodal relapse. As outlined in the aforementioned study [32], the treatment in PP may be inappropriate for patients with positive sentinel lymph node biopsy who did not received a complete axillary dissection or those with otherwise subclinical axillary disease.

In previously published studies, PP-WBI was reported to be disadvantageous for smaller breasts, concerning the dose applied to the heart with an estimated threshold CTV of >496 mL providing a better protection of the heart in PP compared to SP derived from an equivalent uniform dose model in one publication [27] and Δ mean heart dose (SP and DIBH – PP and FB) of -5.8 cGy for breast volume <592.1 mL compared to 1.2 cGy for volumes of 592.1 – 920.3 mL and 13.4 cGy for volumes >920.3 mL, respectively, p=0.05 in another publication [33]. In our analysis, we detected no statistically significant association between breast size represented by the CTV and the Dmean applied to the heart and its right-sided substructures. These results are in line with two previous publications where a Δ mean heart dose (SP – PP) of 0.2 Gy for breast volumes <500 mL was reported compared to -01 to -0.4 Gy for breast volumes >500 mL, p=0.01 [12], and where a maximum heart dose of 35.76 Gy was found for PTVs >1500 mL and 32.28 Gy for PTVs < 1500 mL in PP compared to 40.81 Gy for PTVs > 1500 mL and 42.09 Gy for PTVs < 1500 mL in SP, p=0.003 [31].

Few studies have evaluated patients treated in PP for right-sided breast cancer. In particular, none of the previously published studies investigating WBI in PP for right-sided breast cancer provided data based on technical setups similar to our approach (i.e., hypofractionation, DIBH, and IMRT in SP, FB, and 3D-conformal radiotherapy in PP, CTV-PTV margins in the order of 5 mm). Furthermore, studies investigating WBI in PP, which included a relevant proportion of patients with right-sided cancer, did not report separately about the detailed data for right-sided WBI [31, 34, 35].

Although WBI was performed differently with normofractionated target doses of 50 Gy and no DIBH in SP, we think that the comparison of our results with two previously published studies about the mean doses applied to the heart and right lung provides some meaningful information. A previous study analyzed 30 patients with right-sided tumor who received 50 Gy in a volumetric modulated arc therapy (VMAT) technique, no DIBH in SP, and a 5 mm CTV-PTV margin [27]. Another, large study on right-sided WBI in PP and SP evaluated 146 patients, again without DIBH in SP, but no CTV-PTV margin [36]. Notably, the accidental doses applied to the OAR in this latter study were not presented with mean doses but with mean absolute dose deviations (MADD) [37]. Comparison of the mean doses or MADD, applied to the heart and lung of these two previously published studies, with our findings revealed a better protection of the lung in PP across all three studies, whereas a better protection of the heart in PP was only seen in one study [27] (see Table 6).These differences might be explained at least in part by the different technical setups. Noteworthy, MADD and mean doses are different metrics and our comparison of MADD with mean doses represents an approximation, only.

**Table 6:** Lung and heart doses in prone versus supine position of previous studies and our investigation

Organ at risk	Gao et al. [27] mean doses	Fargier-Bochaton et al. [36] MADD	Our study mean doses
Δ mean/MADD heart (Gy)	− 0.13	0.3	0.3
Δ mean/MADD right lung (Gy)	− 2.89	− 0.34	− 2.1

Δ = difference of the doses (prone position minus supine position) applied to the organs at risk;

MADD, with mean absolute dose deviation

The results of our study are compromised by the limited patient number, the retrospective approach and the different radiotherapy techniques used for PP and SP. Furthermore, our investigation was biased due to the selection criteria for the chosen treatments. We offered the two different radiotherapy techniques and the dual planning to the entire patient cohort, but the patients information process was less well structured. Hence, the patient´s decisions were potentially skewed by various confounding factors. Furthermore, we offered a simultaneously integrated boost for those patients with an indication for boost irradiation to the tumor bed. This was an attractive option leading to a shortened treatment time, but could only be performed in SP at our department. In all, only less than 10% of the patients chose the dual planning option.

Smoking elevates the risk for lung cancer and cardiovascular events and this risk increases after WBI for both events. Our presented estimation of these hazards for PP vs. SP irradiation would indicate even higher risks when calculated for smokers. However, the limitations of this risk model, outlined below, preclude clear recommendations. Hence, smoking history was not a selection criterion for the choice of the patient´s positioning.

The estimation of cardiovascular events and secondary lung cancers was derived from investigations with older radiotherapy techniques and higher WBI doses. Furthermore, the calculated difference of the mean dose applied to the heart (Δ 3D-PP – SP) was only 0.3 Gy. This small difference might exceed the uncertainty of the risk model for cardiovascular events. More accurate estimations should be derived from larger, prospective clinical studies with longer patient follow-up.

Finally, the technical limitation of our RGSC-breathing control system precludes the implementation of DIBH in PP, which would provide a better protection of the heart and its substructures.

## Conclusions

In this study, we present our planning experience of WBI for right-sided breast cancer in PP, performed with FB and 3D plans, compared to SP, performed in DIBH with IMRT plans. Our data revealed a better protection of the right lung in PP, which likely exceeds the disadvantages of higher doses applied to the heart. The dose coverage of the PTVs was significantly better in SP. We detected no statistically significant differences in the dose coverages of the axillary levels in SP compared to PP, except for level II where the dose coverage was significantly lower in PP. The relevance of this difference remains unclear and could be explored by further clinical investigations. The breast size, represented by the CTV volume, had no statistically significant impact on the mean doses applied to the heart, including its right-sided substructures and the right lung in PP. Improved data concerning the protection of lung and heart and the dose coverage of the target volumes could be achieved with the implementation of DIBH and IMRT in PP. At out department, that was precluded due to technical limitation of our RGSC breathing control system.

## Data Availability

The datasets generated during and/or analyzed during the current study are not publicly available due to granted anonymization to participating patients, but are available in an anonymized version from the corresponding author upon reasonable request.

## Abbreviations

3D: Three dimensional
CT: Computed tomography
CTV: Clinical target volume
DIBH: Deep inspiratory breath hold
ESTRO: European society for radiotherapy and oncology
FB: Free breathing
Gy: Gray
IMRT: Intensity modulated radiotherapy
LAD: Left anterior descending coronary artery
MADD: Mean absolute dose deviation
OAR: Organs at risk
PP: Prone position
pTis: Breast cancer in situ
PTV: Planning target volume
RCA: Right coronary artery
RGSC: Respiratory gating for scanners
RTOG: Radiation therapy oncology group
SP: Supine position
VMAT: Volumetric modulated arc therapy
WBI: Whole breast irradiation
